# Tissue Metabolism, Hematotoxicity, and Hepatotoxicity of Trichlorfon in *Carassius auratus gibelio* After a Single Oral Administration

**DOI:** 10.3389/fphys.2018.00551

**Published:** 2018-05-23

**Authors:** Jianfei Lu, Minli Zhang, Liqun Lu

**Affiliations:** ^1^National Pathogen Collection Center for Aquatic Animals, Shanghai Ocean University, Shanghai, China; ^2^Key Laboratory of Freshwater Aquatic Genetic Resources, Ministry of Agriculture, Shanghai Ocean University, Shanghai, China; ^3^National Experimental Teaching Demonstration Center for Fishery Sciences, Shanghai Ocean University, Shanghai, China

**Keywords:** trichlorfon, silver crucian carp, toxicokinetics, biomarkers, histopathology

## Abstract

Trichlorfon is a most widely used organophosphate insecticide in aquaculture, many successful results have been reported for bath treatments of trichlorfon to control parasites. However, immersion treatments of large stocks with trichlorfon has caused serious environmental pollution. In contrast, oral administration treatment has advantages on reducing environmental pollution and having little effect in non-targeted species. The aim of this study was to investigate the effect of trichlorfon on *Carassius auratus gibelio* physiology after a single oral administration. In this study, *Carassius auratus gibelio* was subjected to oral gavage with various concentrations of trichlorfon (0.5 g/kg, 1 g/kg, and 2 g/kg). The trichlorfon concentration in the plasma and liver tissue was quantified using liquid chromatography-tandem mass spectrometry at different time points. At the beginning of oral exposure, the uptake of trichlorfon in the plasma and liver tissue was fast, and trichlorfon was rapidly eliminated to a low level within 24 h. In addition, acetylcholinesterase, superoxide dismutase, catalase, and glutathione-S-transferase activities in the plasma and liver tissue changed significantly after trichlorfon exposure. Additionally, vacuolar degeneration, necrosis, and congestion of the central vein were observed in the liver after trichlorfon exposure, as assessed by hematoxylin and eosin staining. Our results suggested that trichlorfon could accumulate and induce hematotoxicity and hepatotoxicity in the plasma and liver tissue, the toxicity induced by trichlorfon might result in physiological disturbances in fish.

## Introduction

Organophosphate (OP) pesticides are used worldwide to control parasites in agriculture, industry, and households (Terry, 2012; Kalita et al., 2016). The OP trichlorfon, is used to control various parasitic infestations in human medicine, agriculture, and aquaculture industries (WHO, 1992). However, the use of trichlorfon on a large scale has caused serious environmental pollution. In particular, after occupational or accidental exposure to trichlorfon residues, humans have suffered reproductive, mutagenic and carcinogenic toxicity (Fernandes et al., 2015; Yonar et al., 2015). Thus, in some countries such as the United States, trichlorfon is registered for non-agricultural uses, such as animal kennels, ornamental shrubs and plants, and is also registered for indoor non-food use (Chen et al., 2016). However, trichlorfon is still extensively used in agriculture on a global scale, and its product volume is even increasing in developing countries.

Trichlorfon has been widely used in aquaculture, many successful results have been reported for trichlorfon bath treatment of fish infested by *Ergasilus* sp., *Lernea* sp., *Dactylogyrus* sp., *Trichodinas* sp. in freshwater fish (Cruze Silva et al., 2000), against sea lice in salmon farms (Grave et al., 1991), and against *Diplectanum* sp in *Dicentrarchus labrax* (Varriale and Cecchini, 1992; Silan et al., 1996). Immersion treatments of large stocks with trichlorfon have caused serious problems, including environmental pollution, toxicity to the target fish, toxicity to non-targeted species (fishes, crabs, and shrimp), and anthelmintic resistance. The trichlorfon administered orally was also reported in the treatment of *D. aequans* infestations in cultured sea bass (Toksen et al., 2012), and this option has shown advantages, such as reducing environmental pollution and having little effect in non-targeted species.

The primary effect of trichlorfon is the inhibition of the acetylcholinesterase (AChE), which causes accumulation of acetylcholine (ACh) at nerve synapses and disrupts nerve function (Fernandes et al., 2015). Superoxide dismutase (SOD), glutathione S-transferase (GST), and catalase (CAT) are useful biomarkers to describe the integrated toxicological effects of pharmaceuticals (Dröge, 2002). SOD is an important antioxidant enzyme that is involved in the removal of excess superoxide anion free radicals. CAT is also an important antioxidant enzyme that prevents production of the harmful anion OH^-1^, and plays an important role in alleviating oxidative damage (Zhong et al., 2011). The inhibition of AChE activity occurred after prawns were exposed to trichlorfon for 6 h, which caused subsequent acute physiological responses and clinical symptoms (Chang et al., 2013). Trichlorfon also induces oxidative stress, leading to reactive oxygen species (ROS) generation and alterations in antioxidants or free oxygen radicals scavenging enzyme systems in aquatic organisms. The liver of trichlorfon-treated Nile tilapia presented significant increases in CAT and GST activities, and a significant reduction in the SOD activity (Thomaz et al., 2009). With exposure to 1 mg/L and 45 mg/L trichlorfon, the activities of AChE and SOD in the hepatopancreas of *Carassius auratus gibelio* decreased (Wei-Na et al., 2007). Despite previous investigations of trichlorfon, the mechanism of oral trichlorfon-induced toxicity in aquatic animals remains unclear.

The aim of this study was to investigate the effect of trichlorfon on *Carassius auratus gibelio* physiology after an oral administration. Hence, the metabolism kinetics of trichlorfon in *Carassius auratus gibelio* plasma and liver tissues was examined. Furthermore, the AChE, GST, SOD, and CAT activities were determined in the plasma and liver tissue, and liver histopathology was measured to explore the toxic effect of trichlorfon.

## Materials and Methods

### Chemicals and Fish

Trichlorfon (>90% pure) was purchased from Shanghai Biochemical Reagent, Shanghai, China. Healthy silver crucian carp (*Carassius auratus gibelio*; approximately 10 cm in body length) were obtained from the Wujiang National Farm of Chinese Four Family Carps, Jiangsu Province, China. Initially, the fish were reared at 22 ± 2°C in 400 L aerated tanks for 1 week before the experiment and fed twice daily (in the morning and late in the afternoon) at a ratio of 5% of their total biomass. After 7 days of acclimation, fish were starved for 2 days before the administration of the drug. All experiments were performed according to the guidance of the Care and Use of Laboratory Animals in China. This study was approved by the Committee on the Ethics of Animal Experiments of Shanghai Ocean University, China.

### Experimental Design

All the fish were divided into four groups (40 fish per group). The conditions were identical among the tanks and the fish were randomly distributed into the different tanks. To prepare the trichlorfon dosing solution, trichlorfon was dissolved in 5 mL of ethanol and mixed with water to achieve a final concentration of 2 g/L. The fish were given 2 g/L of the trichlorfon solution via gavage using a stomach tube, with final trichlorfon doses of 0.5 g/kg, 1 g/kg, or 2 g/kg. As controls, fish were given ethanol mixed with water at the same dosage. After oral administration, each fish was placed in an observation tank for 5 min to check for possible drug regurgitation; regurgitating fish were excluded from the analysis.

### Sample Collection

Three fish of each group were anesthetized with 2-phenoxyethanol (2 mL/L) before handling, and sampled at 1, 2, 3, 4, 8, 12, 24, 48, 72, and 96 h after the oral drug administration. For the enzymatic analysis, blood was taken from the caudal vein, incubated at 37°C for 1 h, stored at 4°C for 12 h, and centrifuged at 2000 × *g* for 10 min in 4°C, and the plasma supernatant was collected for the later experiments. Hepatocyte tissues were washed with ice-cold saline (0.85% NaCl), homogenized in (1:9 w/v) ice-cold 0.1 M pH 7.4 phosphate buffer using a glass homogenizer, and the homogenate was centrifuged for 20 min (10000 × *g*) at 4°C. The supernatant was used as the enzyme source to assess the enzyme activities. All the preparations were frozen at -80°C until analysis.

### Sample Preparation and Extraction

One milliliter of blood was taken from the caudal vein at 1, 2, 3, 4, 8, 12, 24, 48, 72, and 96 h after oral drug administration and mixed with 14 mL of ethyl acetate. Then, 2 g of hepatocyte tissues were homogenized in centrifuge tube, and 15 mL of ethyl acetate was added. The slurry was vortexed for 1 min. The samples were centrifuged at 2,292 × *g* for 5 min, and the clear supernatant was collected and transferred into a new 50 mL centrifuge tube. The extraction procedure was repeated twice with 10 mL of the ethyl acetate. The supernatants were combined and concentrated to 5 mL under a stream of nitrogen at 40°C. The residues were extracted using Copure Alumina SPE (Biocomma, Shenzhen, China), and dissolved in 5 mL of ethyl acetate. Finally, the extract was evaporated to near dryness under a stream of nitrogen at 40°C, dissolved in 1.0 mL of the mobile phase (65% water, 35% methanol, and 0.1% of formic acid). The extract was filtered using a 0.22-μm nylon filter for liquid chromatography-tandem mass spectrometry (LC–MS/MS) analysis. All samples were analyzed in duplicate.

### LC–MS/MS Analysis

Samples were analyzed on an LTQ-Orbitrap XL instrument (Thermo Scientific, Bremen, Germany). The parameters were as follows: Mobile phase A: water containing 0.1% of formic acid (v/v); Mobile phase B: methanol; injection volume 2 μL; mobile phase flow rate 0.2 mL/min; mobile phase gradients: 35% B (0–6.00 min), 95% B (6.01–10.00 min), and 35% B (10.01–14.00 min); electrospray positive ionization (ESI+); gas flow was set at 10 L/min; gas temperature was set at 350°C; nebulizer pressure 25 psi; capillary voltage 3500 V, gas temperature 350°C; nebulizer pressure 50 psi; capillary voltage 5500 V. The multiple reaction monitoring (MRM) mode was positive, and the optimized MS/MS transitions were m/z 259.1>221.1.

### AChE Activity

Acetylcholinesterase activity was quantified according to the colorimetric technique as previously described (Ellman et al., 1961). All samples were analyzed in triplicate at 25°C. Acetylthiocholine iodide (ATC) was used as the substrate and 5,5′-Dithiobis (2-nitrobenzoic acid) (DTNB dissolved in phosphate buffer at pH 7.0) was used as the color reagent. Finally, the optical density was determined using a SPECTRA MAX 190 spectrophotometer (Molecular Devices, United States) at 415 nm. AChE activity was expressed as various levels of absorbance per **mg protein in** hepatocytes and per ml in plasma.

### SOD, CAT, and GST Activities

The activities of SOD, CAT, and GST were quantified using commercial kits (Jiancheng, Nanjing, China) according to the manufacturer’s instructions. The total protein concentration of the samples was determined at 595 nm using the Bradford method. GST activity was assayed by measuring the formation of GSH (Glutathione) and the 1-chloro-2,4-dinitrobenzene (CDNB) conjugate. The specific activity of GST was expressed as U/mg of protein in hepatocytes and U/mL in plasma. SOD activity was measured at 550 nm, one unit of SOD activity was defined as the amount of enzyme required to inhibit the oxidation reaction by 50% and was expressed as U/mg of protein in hepatocytes and U/mL in plasma. CAT activity was determined by assaying the hydrolysis of H_2_O_2_ and the resulting decrease in absorbance at 405 nm over a 3 min period at 25°C. One unit of CAT activity was defined as the amount of enzyme required to consume 1 mol of H_2_O_2_ in 1 s and was expressed as U/mg of protein in hepatocytes and U/mL in plasma.

### Histopathological Examination

For histopathological examination, three fish from each group per replicate for histopathological studies were removed after treatment periods of 24 h. Hepatocyte tissues were dissected and fixed in Bouin’s fluid for 24 h, and washed with tap water. They were processed through a graded ethanol series, cleared in xylene, and embedded in paraffin. Paraffin samples were sectioned at a thickness of 5 μm, and stained with hematoxylin and eosin (HE) for light microscopic observation. The sections were examined under a light microscope (Olympus Vanox photomicroscope, Japan).

### Data Analyses

Data was tested for normality and homogeneity of variance before statistical analysis. All data analyses were carried out using IBM SPSS Statistics Version 20. One-way analysis of variance (ANOVA) was used to test for differences, followed by the Tukey test (*post hoc* test). All the results are presented as mean ± SD of three independent experiments. The results of one-way ANOVAs are denoted as ^∗^ for *P* < 0.05 and ^∗∗^ for *P* < 0.01.

## Results

### Metabolism of Trichlorfon in Plasma and Liver Tissue

**FIGURE 1:**
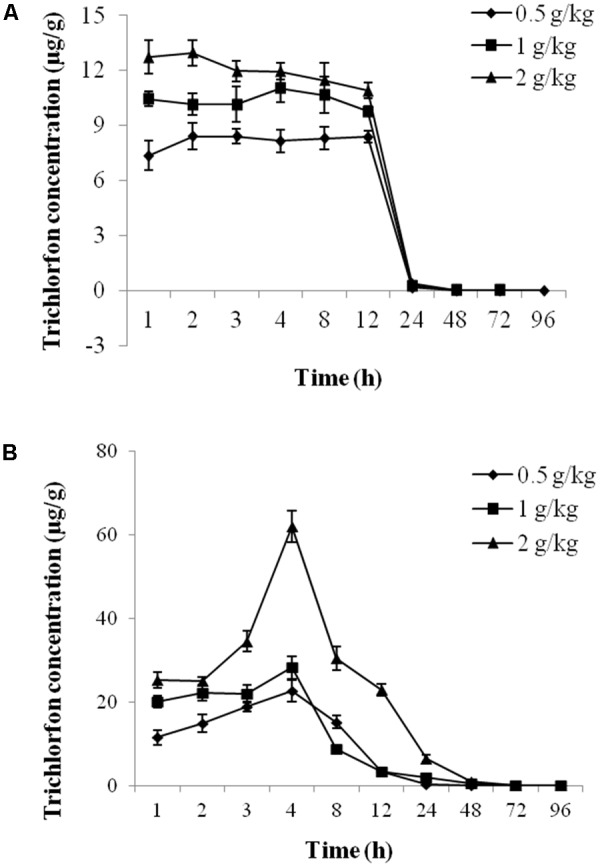
The concentration-time profile of trichlorfon in *Carassius auratus gibelio* plasma and liver tissue after oral exposure. **(A)** Plasma; **(B)** liver (*n* = 3, data are shown as the mean ± SD).

The concentration-time profiles of plasma and liver tissue after an oral dose of 0.5 g/kg, 1 g/kg, and 2 g/kg of trichlorfon are shown in **Figure 1**. The changes in the concentrations of trichlorfon in the plasma and liver tissue were time and dose-dependent. At the beginning of oral exposure, the uptake of trichlorfon in the plasma and liver tissue was fast, after which trichlorfon was rapidly eliminated to a low level within 24 h. The peak levels (Cmax) in plasma were 9.793, 11.715, and 13.208, and the Cmax in liver were 20.495, 24.912, and 45.058 (0.5 g/kg, 1 g/kg, and 2 g/kg, respectively). Additionally, the high dose (2 g/kg) resulted in a shorter time (3.56 h) to peak level compared with the lower dose (5.37 h for 0.5 g/kg) in plasma, and in liver tissue, the shortest time to peak level was 2.65 h at a dose of 1 g/kg (**Table 1**).

**Table 1:** Pharmacokinetic parameters of TCF in *Carassius auratus gibelio* (*n* = 3).

Parameter	Unit	Plasma			Liver		
		0.5 mg/kg	1 mg/kg	2 mg/kg	0.5 mg/kg	1 mg/kg	2 mg/kg
Cmax	μg/g	9.793	11.715	13.208	20.495	24.912	45.058
Tmax	h	5.37	4.75	3.56	3.69	2.65	4.45
T_1/2alpha_	h	5.554	4.169	5.143	2.302	0.123	2.076
T_1/2Ka_	h	2.796	4.681	3.941	2.535	1.808	4.461
Kel	h	0.133	0.758	1.102	0.796	0.583	0.208
CLs	L/h⋅kg	6.520	10.229	18.664	5.158	10.494	7.088
AUC	μg/L⋅h	153.363	195.523	214.317	193.884	190.592	564.314
Ka	1/h	0.248	0.148	0.176	0.273	0.383	0.155

*Cmax, the peak concentration in plasma; Tmax, the time point of the drug’s maximum plasma concentration; T_*1/2 alpha*_, distribution half-life of the drug; T_*1/2 Ka*_, absorption half-life of the drug; Kel, elimination rate constant from the central compartment; CLs, total body clearance; AUC, Area under the drug concentration-time curve from the time zero to last time point; Ka, absorption rate constant.*

### AChE Activity

**FIGURE 2:**
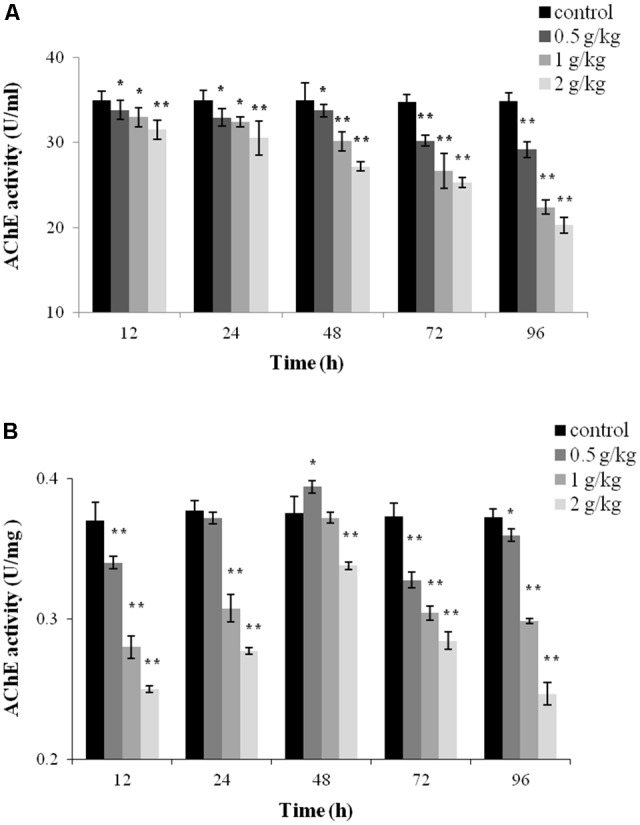
Acetylcholinesterase (AChE) activity in *Carassius auratus gibelio* after a single dose of 0.5 g/kg, 1 g/kg, or 2 g/kg trichlorfon. **(A)** Changes in AChE activity in plasma after a single dose of trichlorfon; **(B)** changes in AChE activity in liver tissues after a single dose of trichlorfon. Asterisks indicate values that are significantly different from the control values, ^∗^*P* < 0.05; ^∗∗^*P* < 0.01, *n* = 3.

Acetylcholinesterase activity in the plasma and liver tissue was monitored after a single dose of 0.5 g/kg, 1 g/kg, or 2 g/kg of trichlorfon. As shown in **Figure 2A**, a significant decrease of AChE activity was observed in all tested samples after oral administration of trichlorfon in the plasma, and this reduction was concentration and time-dependent; a higher dose of trichlorfon resulted in lower AChE activity. In liver tissues, AChE activity was significantly reduced at 12 h after oral administration (*P* < 0.01), and upregulated at 24 and 48 h post oral administration, followed by a gradual decrease at 72 and 96 h (**Figure 2B**).

### GST Activity

**FIGURE 3:**
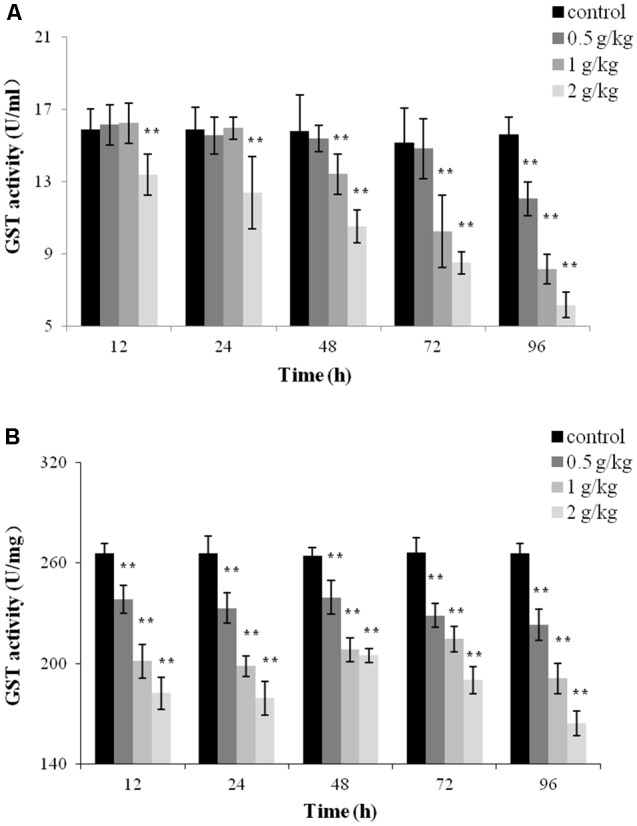
Glutathione S-transferase (GST) activity of *Carassius auratus gibelio* after a single dose of 0.5 g/kg, 1 g/kg, or 2 g/kg trichlorfon. **(A)** Changes in GST activity in plasma after a single dose of trichlorfon; **(B)** changes in GST activity in liver tissues after a single dose of trichlorfon. Asterisks indicate values that are significantly different from the control values, ^∗^*P* < 0.05; ^∗∗^*P* < 0.01, *n* = 3.

To evaluate the toxicity of trichlorfon, GST activity was detected after a single dose of 0.5 g/kg, 1 g/kg, or 2 g/kg of trichlorfon in the plasma and liver tissues. At a dose of 0.5 g/kg, the activity of GST only decreased at 96 h post oral administration in the plasma (*P* < 0.01); at 1 g/kg, the activity of GST was significantly downregulated at 48, 72, and 96 h (*P* < 0.01); at 2 g/kg, the activity of GST gradually decreased in a time-dependent manner (**Figure 3A**). However, in liver tissues, significant decreases in GST activity was observed at all tested samples after oral administration, and this reduction was concentration and time-dependent.

### CAT and SOD Activities

**FIGURE 4:**
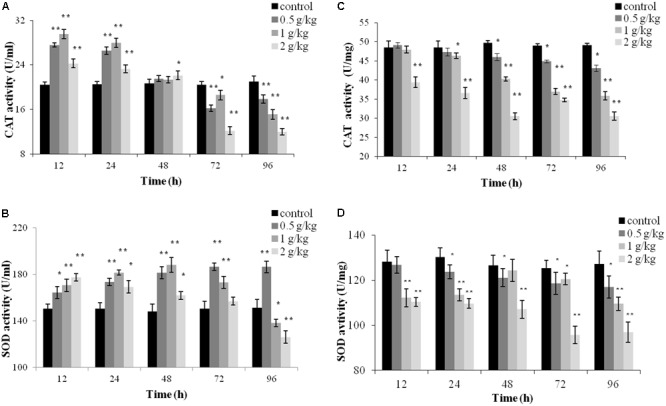
Catalase (CAT) and superoxide dismutase (SOD) activities of *Carassius auratus gibelio* after a single dose of 0.5 g/kg, 1 g/kg, or 2 g/kg trichlorfon. **(A)** Changes in CAT activity in plasma after a single dose of trichlorfon; **(B)** changes in SOD activity in plasma after a single dose of trichlorfon; **(C)** changes in CAT activity in liver tissues after a single dose of trichlorfon; and **(D)** changes in SOD activity in liver tissues after a single dose of trichlorfon. Asterisks indicate values that are significantly different from the control values, ^∗^*P* < 0.05; ^∗∗^*P* < 0.01, *n* = 3.

Catalase and SOD activities in the plasma and liver tissues post oral administration are presented in **Figure 4**. In the plasma, significant increased of CAT activity was observed at all tested concentrations of trichlorfon at 12 and 24 h (*P* < 0.01), and significant increased of CAT activity was only found in dosing of 2 g/kg at 48 h (*P* < 0.05). However, CAT activity induction decreased with exposure time, and CAT was significantly suppressed at 72 and 96 h post oral administration (**Figure 4A**). SOD activity increased at 12, 24, and 48 h at all tested concentrations of trichlorfon, and was inhibited at doses of 1 g/kg and 2 g/kg at 96 h post oral administration (**Figure 4B**).

In liver tissues, CAT activity was inhibited in a dose-dependent manner. At a dose of 0.5 g/kg, CAT activity was reduced at 48 h (*P* < 0.01), followed by a gradual decrease at 72 and 96 h (*P* < 0.01); at 1 g/kg, CAT activity was reduced at 24 h (*P* < 0.05), followed by a gradual decrease at 48, 72, and 96 h (*P* < 0.01); and at 2 g/kg, CAT activity was reduced from 12 h (*P* < 0.01) (**Figure 4C**). At a dose of 0.5 g/kg, SOD activity was reduced at 24 h (*P* < 0.05), followed by a gradual decrease at 48, 72, and 96 h (*P* < 0.05). At doses of 1 g/kg and 2 g/kg, SOD activity was reduced after 12 h (*P* < 0.01) (**Figure 4D**).

### Histopathological Changes

**FIGURE 5:**
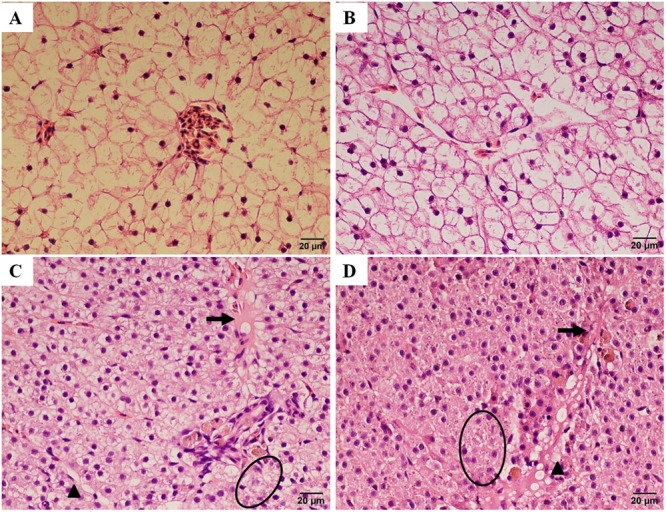
Histopathological changes in the liver of *Carassius auratus gibelio* after treatment with trichlorfon for 24 h. **(A)** Liver of control fish; **(B)** liver of *Carassius auratus gibelio* after oral administration of 0.5 g/kg trichlorfon; **(C)** oral administration of 1 g/kg trichlorfon; and **(D)** oral administration of 2 g/kg trichlorfon. Vacuolar degeneration (triangle), necrosis (circle), and congestion of the central vein (arrow).

A histopathological examination was carried out to determine the extent of hepatotoxicity as a consequence of trichlorfon treatment in liver tissue. As illustrated in **Figure 5A**, the liver in the control group presented normal features and showed obvious cell nuclei, sinusoids, and delimited cytoplasm. At a dose of 0.5 g/kg of trichlorfon, no obvious differences in the liver sections were observed compared with the control group (**Figure 5B**). However, at 1 g/kg, vacuolar degeneration, necrosis, and congestion of the central vein were observed in the liver tissues (**Figure 5C**). Similar phenomena were observed in the liver tissues of the 2 g/kg group (**Figure 5D**). These results of HE staining in liver tissues suggested that the orally administered of trichlorfon induced hepatic injury.

## Discussion

It is reported that trichlorfon causes many physiological changes in experimental animals (Falfushynska et al., 2012). In this study, tissue metabolism, hematotoxicity, and hepatotoxicity changes in *Carassius auratus gibelio* were examined after a single oral administration of trichlorfon. At the beginning of oral exposure, the uptake of trichlorfon in the plasma and liver tissue was fast, after which trichlorfon was rapidly eliminated to a low level within 24 h. In addition, the AChE, SOD, CAT, and GST activities in the plasma and liver tissues changed significantly after trichlorfon exposure, although the exact mechanisms underlying the toxicity responses are not clear. Finally, we detected the effect of trichlorfon on liver tissue using HE staining.

Acetylcholinesterase is mainly responsible for the digestion of the neurotransmitter ACh in the synaptic cleft (Fernandes et al., 2015). There are several reports of AChE activity inhibition in aquatic animal exposed to trichlorfon. Cultivated Nile Tilapia exposed to trichlorfon showed a significant suppression of AChE activity in muscle tissue (Guimarães et al., 2007). The activity of AChE was reduced after exposure to trichlorfon in white muscle and brain of freshwater fish pacu (Venturini et al., 2015). In the present study, a significant decrease of AChE activity was observed in the plasma and liver tissue after a single dose of trichlorfon (**Figure 2**). The persistent suppression of AChE can inhibit fish activities (e.g., swimming) (Tierney et al., 2007; Pereira et al., 2012). ACh persistence in the synaptic cleft leads to continuous stimulation of muscle or nerve fibers, resulting in exhaustion and tetany (Fukuto, 1990). Continuous suppression of AChE might damage the organism’s ability to search for food and shelter, and inhibit cohort behavior and escape from predators.

Glutathione S-transferase is an important member of a supergene family that is involved in protecting against the deleterious effects of xenobiotics and oxidative stress (Ma et al., 2017). In a previous study, a decrease in GST activity was observed in the tissues of *Cyprinus carpio* exposed to bispyribac-sodium under field conditions (Toni et al., 2010). Similarly, the suppression of GST activity in liver were also observed in *Carassius auratus gibelio* exposed to thiocarbamate Tatoo or tetrazine Apollo (Falfushynska et al., 2012). Our study found that the GST activity was inhibited in the plasma and liver tissue after a single dose of trichlorfon (**Figure 3**). Downregulation of GST activity highlighted the fact that GST probably plays crucial physiological roles during trichlorfon detoxification in *Carassius auratus gibelio*.

Superoxide dismutase and CAT are important antioxidant enzymes, and are scavengers of ROS. The activation of SOD and CAT constitutes the first line of defensive against oxidative stress (Ken et al., 2005; Singh et al., 2016). SOD catalyzes the conversion of superoxide radicals to hydrogen peroxide, while CAT converts hydrogen peroxide into water (Mansour and Mossa, 2009). Trichlorfon has been reported to induce oxidative stress in the hearts of Nile Tilapia (Thomaz et al., 2009). In this study, the increase of SOD and CAT activities in the plasma of *Carassius auratus gibelio* following trichlorfon treatment might have resulted from increased formation of reactive oxygen, which could stimulate CAT and SOD activities. However, the CAT activity was significantly inhibited at 72 and 96 h, and SOD was decreased at doses of 1 g/kg and 2 g/kg at 96 h. This trend (initial elevation followed by a decrease) of anti-oxidase levels has also been observed in zebrafish hepatocytes exposed to beta-cypermethrin, and could be a signal of an overwhelmed antioxidant capacity caused by long-term exposure (Dogan et al., 2011). Contrary to the results in the plasma, SOD and CAT activities in the liver were inhibited. CAT activity was inhibited by trichlorfon in liver of Zebra fish after exposure to 5, 10, and 20 mg/L trichlorfon (Coelho et al., 2011). Inhibition of SOD and CAT activities was also observed in rat exposed to Chlorpyrifos (Uzun and Kalender, 2013). It has been proved that pesticides can cause tissue-specific responses (Oruc et al., 2004; Durmaz et al., 2006), which could explain, at least in part, the different results acquired in *Brycon cephalus* when compared with those in our study (Monteiro et al., 2006).

Histopathological assays have been widely used as indices for the investigations of pesticide toxicity (Uzun et al., 2009). OP pesticides induce various histopathological changes in aquatic animal (Gokalp et al., 2003; Gokcimen et al., 2007; Sayim, 2007). Several histopathological changes to the gills of tilapia were observed after exposure to trichlorfon (Guimarães et al., 2007). Trichlorfon exposure to low sublethal doses for 4 and 8 weeks in wistar rats led to cell damage in astrocytes and neurons in the hippocampus and striatum (Hernández et al., 2013). In this study, no obvious differences in the liver sections compared with the control group at a dose of 0.5 g/kg were observed. Vacuolar degeneration, necrosis, and congestion of the central vein were observed at doses of 1 and 2 g/kg (**Figure 5**). These results were similar to those obtained in tilapia, which indicated that low concentrations of trichlorfon did not cause significant alterations in the tissue (Guimarães et al., 2007). However, high concentrations or long time treatment with low concentrations of trichlorfon might increase the pathological severity, consequently compromising animal health (Venkateswara et al., 2003). Besides, AChE, GST, SOD, and CAT are useful biomarkers to describe the integrated toxicological effects of pharmaceuticals. In this study, AChE, GST, SOD, and CAT activity were significantly inhibited following trichlorfon treatment in liver tissues, the inhibition of enzyme activity may leaded to hepatotoxicity, and the hepatotoxicity expressed as histopathological changes.

Overall, by combining analysis of the metabolism and toxicity of trichlorfon in the plasma and liver post oral exposure, we investigated the hematotoxicity and hepatotoxicity of trichlorfon in *Carassius auratus gibelio*. The exposure concentration played an important role in the bioconcentration of trichlorfon, and its increase could significantly affect the uptake rates, Cmax and Tmax. AChE, GST, SOD, and CAT activity were significantly inhibited following trichlorfon treatment in liver tissues. AChE and GST activity in plasma were inhibited by trichlorfon, and SOD and CAT activity were increased by trichlorfon. Additionally, high oral concentration of trichlorfon induced histopathological changes in liver tissues, including vacuolar degeneration, necrosis, and congestion of the central vein. Considering the toxicity of trichlorfon on *Carassius auratus gibelio*, there should be a serious apprehension about the potential danger of this pesticide on fish. Therefore, further work should be performed to determine the precise physiological action and molecular mechanism of trichlorfon in fish.

## Author Contributions

LL designed and led the study. MZ and JL performed the experiments. JL drafted the manuscript. All authors read and approved the final version of the manuscript.

## Conflict of Interest Statement

The authors declare that the research was conducted in the absence of any commercial or financial relationships that could be construed as a potential conflict of interest.

## References

[B1] Chang C. C. Rahmawaty A. Chang Z. W. (2013). Molecular and immunological responses of the giant freshwater prawn, *Macrobrachium rosenbergii*, to the organophosphorus insecticide, trichlorfon. 18 130–131. 10.1016/j.aquatox.2012.12.024 23340335

[B2] Chen X. J. Ren Y. J. Meng Z. Y. Lu C. L. Gu H. T. Zhuang Y. Q. (2016). Dissipation kinetics, safety evaluation, and preharvest interval assessment of trichlorfon application on rice. 188 1–9. 10.1007/s10661-016-5264-9 27048491

[B3] Coelho S. Oliveira R. Pereira S. Musso C. Domingues I. Bhujel R. C. (2011). Assessing lethal and sub-lethal effects of trichlorfon on different trophic levels. 103 191–198. 10.1016/j.aquatox.2011.03.003 21473847

[B4] Cruze Silva M. P. Orge M. L. Afonso-Roque M. M. Grazina-Freitas M. S. Carvalho-Varela M. (2000). *Diplectanum aequans* (Wagener, 1857) Diesing, 1858 (Monogenea, Diplectanidae) in sea bass (*Dicentrarchus labrax* (L.), 1758) from freshwater culture. 7 53–56.

[B5] Dogan D. Can C. Kocyigit A. Dikilitas M. Taskin A. Bilinc H. (2011). Dimethoate-induced oxidative stress and DNA damage in *Oncorhynchus mykiss*. 84 39–46. 10.1016/j.chemosphere.2011.02.087 21435680

[B6] Dröge W. (2002). Free radicals in the physiological control of cell function. 82 47–95. 10.1152/physrev.00018.2001 11773609

[B7] Durmaz H. Sevgiler Y. Üner N. (2006). Tissue-specific antioxidative and neurotoxic responses to diazinon in *Oreochromis niloticus*. 84 215–226. 10.1016/j.pestbp.2005.07.004

[B8] Ellman G. L. Courtney K. D. Andres V. Jr. Featherstone R. M. (1961). A new and rapid colorimetric determination of acetylcholinesterase activity. 7 88–95. 10.1016/0006-2952(61)90145-9 13726518

[B9] Falfushynska H. I. Gnatyshyna L. L. Stoliar O. B. (2012). Population-related molecular responses on the effect of pesticides in *Carassius auratus gibelio*. 155 396–406. 10.1016/j.cbpc.2011.11.001 22119335

[B10] Fernandes L. S. Emerick G. L. Santos N. A. de Paula E. S. Barbosa F. B. F. Jr. Santos A. C. (2015). In vitro study of the neuropathic potential of the organophosphorus compounds trichlorfon and acephate. 29 522–528. 10.1016/j.tiv.2015.01.001 25596135

[B11] Fukuto T. R. (1990). Mechanism of action of organophosphorus and carbamate insecticides. 87 245–254. 10.1289/ehp.9087245 PMC1567830 2176588

[B12] Gokalp O. Gulle K. Sulak O. Cicek E. Altuntas I. (2003). The effects of methidathion on liver: role of vitamins E and C. 19 63–67. 10.1191/0748233703th176oa 15697176

[B13] Gokcimen A. Gulle K. Demirin H. Bayram D. Kocak A. Altuntas I. (2007). Effects of diazinon at different doses on rat liver and pancreas tissues. 87 103–108. 10.1016/j.pestbp.2006.06.011

[B14] Grave K. Engelstad M. Søli N. E. Toverud E. L. (1991). Clinical use of dichlorvos (Nuvan) and trichlorfon (Neguvon) in the treatment of salmon louse, *Lepeophtheirus salmonis*. Compliance with the recommended treatment procedures. 32 9–14. 1950856 10.1186/BF03546992 PMC8127880

[B15] Guimarães A. T. Hc S. D. A. Boeger W. (2007). The effect of trichlorfon on acetylcholinesterase activity and histopathology of cultivated fish *Oreochromis niloticus*. 68 57–62. 10.1016/j.ecoenv.2006.08.005 17055053

[B16] Hernández Y. T. Barragán I. R. Rubio A. C. (2013). Neurotoxic potential of trichlorfon to multiple sublethal doses in wistar rats. 18 479–487.

[B17] Kalita M. K. Haloi K. Devi D. (2016). Larval exposure to chlorpyrifos affects nutritional physiology and induces genotoxicity in silkworm *Philosamia ricini* (Lepidoptera: Saturniidae). 7:535. 10.3389/fphys.2016.00535 27895594 PMC5108804

[B18] Ken C. F. Hsiung T. M. Huang Z. H. Juang R. H. Lin C. T. (2005). Characterization of Fe/Mn-Superoxide dismutase from diatom *Thallassiosira weissflogii*: cloning, expression, and property. 53 1470–1474. 10.1007/s10661-016-5264-9 15740026

[B19] Ma Y. Li B. Ke Y. Zhang Y. Zhang Y. (2017). Transcriptome analysis of *Rana chensinensis* liver under trichlorfon stress. 147 487–493. 10.1016/j.ecoenv.2017.09.016 28910747

[B20] Mansour S. A. Mossa A. T. H. (2009). Lipid peroxidation and oxidative stress in rat erythrocytes induced by chlorpyrifos and the protective effect of zinc. 93 34–39. 10.1016/j.pestbp.2008.09.004

[B21] Monteiro D. A. de Almeida J. A. Rantin F. T. Kalinin A. L. (2006). Oxidative stress biomarkers in the freshwater characid fish, *Brycon cephalus*, exposed to organophosphorus insecticide Folisuper 600 (methyl parathion). 143 141–149. 10.1016/j.cbpc.2006.01.004 16546452

[B22] Oruc E. O. Sevgiler Y. Uner N. (2004). Tissue-specific oxidative stress responses in fish exposed to 2,4-D and azinphosmethyl. 137 43–51. 10.1016/j.cca.2003.11.006 14984703

[B23] Pereira V. M. Bortolotto J. W. Kist L. W. Azevedo M. B. D. Fritsch R. S. Oliveira R. D. L. (2012). Endosulfan exposure inhibits brain AChE activity and impairs swimming performance in adult zebrafish (*Danio rerio*). 33 469–475. 10.1016/j.neuro.2012.03.005 22459995

[B24] Sayim F. (2007). Dimethoate-induced biochemical and histopathological changes in the liver of rats. 59 237–243. 10.1016/j.etp.2007.05.008 17869494

[B25] Silan P. Birgi E. Louis C. Clota F. Mathieu A. Giral L. (1996). Aquaculture et ichtyoparasitologie: action in vitro du nitroxinil (anthelminthique) sur Diplectanum aequans, monogene ectoparasite branchial du bar *Dicentrarchus labrax*. 172 401–407.

[B26] Singh D. Cho W. C. Upadhyay G. (2016). Drug-Induced liver toxicity and prevention by herbal antioxidants: an overview. 6(Pt 2):363. 10.3389/fphys.2015.00363 26858648 PMC4726750

[B27] Terry A. V. Jr. (2012). Functional consequences of repeated organophosphate exposure: potential non-cholinergic mechanisms. 134 355–365. 10.1016/j.pharmthera.2012.03.001 22465060 PMC3366364

[B28] Thomaz J. M. Martins N. D. Monteiro D. A. Rantin F. T. Kalinin A. L. (2009). Cardio-respiratory function and oxidative stress biomarkers in Nile tilapia exposed to the organophosphate insecticide trichlorfon (NEGUVON). 72 1413–1424. 10.1016/j.ecoenv.2008.11.003 19171380

[B29] Tierney K. B. Singh C. R. Ross P. S. Kennedy C. J. (2007). Relating olfactory neurotoxicity to altered olfactory-mediated behaviors in rainbow trout exposed to three currently-used pesticides. 81 55–64. 10.1016/j.aquatox.2006.11.006 17145086

[B30] Toksen E. Nemli E. Koyuncu E. Cankurt M. (2012). Effect of trichlorfon on Diplectanum aequans (Monogenea: Diplectanidae) infestations in cultured sea bass, *Dicentrarchus labrax*. 32 495–498.

[B31] Toni C. de Menezes C. C. Loro V. L. Clasen B. E. Cattaneo R. Santi A. (2010). Oxidative stress biomarkers in Cyprinus carpio exposed to commercial herbicide bispyribac-sodium. 30 590–595. 10.1002/jat.1530 20809548

[B32] Uzun F. G. Kalender S. Durak D. Demir F. Kalender Y. (2009). Malathion-induced testicular toxicity in male rats and the protective effect of vitamins C and E. 47 1903–1908. 10.1016/j.fct.2009.05.001 19442699

[B33] Uzun F. G. Kalender Y. (2013). Chlorpyrifos induced hepatotoxic and hematologic changes in rats: the role of quercetin and catechin. 55 549–556. 10.1016/j.fct.2013.01.056 23402859

[B34] Varriale A. M. C. Cecchini S. (1992). Therapeutic trials against the Diplectanum aequans (Monogenea), parasite of seabass (*Dicentrarchus labrax*, L.) in intensive farming. 12 204–206.

[B35] Venkateswara R. J. Shilpanjali D. Kavitha P. Madhavendra S. S. (2003). Toxic effects of profenofos on tissue acetylcholinesterase and gill morphology in a euryhaline fish, *Oreochromis mossambicus*. 77 227–232. 10.1007/s00204-002-0432-9 12698238

[B36] Venturini F. P. Moraes F. D. Cortella L. R. Rossi P. A. Cruz C. Moraes G. (2015). Metabolic effects of trichlorfon (Masoten^®^) on the neotropical freshwater fish pacu (*Piaractus mesopotamicus*). 41 299–309. 10.1007/s10695-014-9983-y 25192665

[B37] Wei-Na X. U. Zhang X. Liu W. B. (2007). Toxicity of trichlorfon against *Carassais auratus* gibebio and its impacting factors. 20 38–39.

[B38] WHO (1992). Geneva: World Health Organization 14.

[B39] Yonar M. E. Yonar S. M. Pala A. Silici S. Saðlam N. (2015). Trichlorfon-induced haematological and biochemical changes in *Cyprinus carpio*: ameliorative effect of propolis. 114 209–216. 10.3354/dao02866 26036828

[B40] Zhong P. Wang Y. F. Ping L. I. (2011). Protective action and its mechanism of polysaccharides on immunological liver injury. 21:3881.

